# Death from Fright

**Published:** 1892-07

**Authors:** 


### DEATH FROM FRIGHT.

There comes a story from Kansas of the sudden and unexpected death of one who, for a slight offense, was made the victim of a mock trial, sentence and execution.

That which was intended to be only a rather cruel practical joke, turned out to be serious calamity. The victim listened to his sentence with fear and trembling, and found no pity in the eyes of his accusers. He was as he verily believed, to be blindfolded and shot from behind his back. Silently and solemnly the preparations were made and at a given signal a piece was discharged in his rear, and at the same instant he was struck sharply with a stick on the back of his head, when to the horror of his tormentors, he fell forward and almost immediately expired. This was all of it, but it was more than enough. The case, however strange is not without others of a similar outcome. Some years ago a like experiment was made upon a prisoner under sentence of death. If our recollection is not at fault, it was in a French prison. The culprit was told that he was to be bled to death. Accordingly he was bound to a stretcher, a slight and bloodless puncture made in one of his arms from the region of which water was caused to drip regularly and audibly into a near-by vessel. Gradually the strength of the victim gave way; fainter and fainter he grew till he swooned and died under the operation.

Thus it may be seen to what an alarming extent we are the creatures of our own imaginings. Our forebodings of evil are oftentimes the open door to like agencies that stand ever ready to enter and do us mischief. On the other hand, if we keep the good always to the front, only the good can enter. Is not this, after all, the secret of the faith cure which has had such a run of late? "As a man thinketh so is he" was not spoken unwittingly nor in vain.

